# Development of Freshness Indicator (FI) for Skate Sashimi (*Zearaja chilensis*) to Detect Trimethylamine Content During Storage

**DOI:** 10.3390/bios15100659

**Published:** 2025-10-02

**Authors:** Kyung-Jik Lim, Yoon-Gil Kim, Yu-Jin Heo, Han-Seung Shin

**Affiliations:** Department of Food Science and Biotechnology, Dongguk University-Seoul, 32, Goyang 10326, Republic of Korea; kyung9209@naver.com (K.-J.L.); rladbsrlf123@naver.com (Y.-G.K.); pdp0616@naver.com (Y.-J.H.)

**Keywords:** smart packaging, freshness indicator (FI), skate sashimi, volatile basic nitrogen (VBN), trimethylamine (TMA)

## Abstract

The seafood industry is increasingly adopting intelligent packaging to preserve product quality and improve freshness transparency. This study developed and evaluated a pH-sensitive freshness indicator (FI) for skate sashimi (*Zearaja chilensis*). This product is consumed at varying stages of fermentation. The FI incorporated bromothymol blue (BTB) and bromocresol purple (BCP) in a polymer matrix. It targeted volatile basic nitrogen (VBN) compounds, with trimethylamine (TMA) as the primary marker. As freshness declined, VBN compounds accumulated in the package headspace and caused a gradual FI color change from yellow to blue through pH variation. Δ*E* increased from 7.72 on day 2 to 23.52 on day 3. This marked the onset of visible color change and the FI reached full blue by day 7. Headspace solid-phase microextraction (HS-SPME) and gas chromatography–flame ionization detection (GC-FID) quantified monomethylamine (MMA), dimethylamine (DMA) and TMA throughout storage. ΔE correlated strongly with total bacterial count (TBC, *r* = 0.978), pH (*r* = 0.901) and TMA (*r* = 0.888). These results indicate that microbial growth, alkalinity increase and amine production were closely associated with color transitions. The FI reliably tracked freshness loss in skate sashimi. It has potential to enhance consumer transparency and strengthen quality control in the seafood supply chain.

## 1. Introduction

Smart packaging is designed to maintain product quality, extend preservation, and provide information [1]. This study focuses specifically on the intelligent type, which is capable of monitoring changes in a product or its surrounding environment and transmitting this information to consumers [2]. Such systems operate by sensing, detecting, and recording environmental changes, thereby enabling direct communication with users. Types of intelligent packaging include indicators and sensors. Indicators are further classified into time-temperature integrators (TTI), freshness indicators (FI), and enzymatic indicators (EI) [3].

FI are cost-effective and compact. They detect changes in the concentration of gases such as oxygen, carbon dioxide, and nitrogenous compounds inside the packaging. These changes appear as color transitions. The color change directly reflects the product’s quality status [4]. In contrast, TTI rely only on the time–temperature history to provide an indirect estimate of freshness. As a result, their response may not always match the actual quality changes in the product [2]. Current FI types are categorized based on the specific target gas they detect. Many pH-sensitive indicators have been reported, particularly film-type systems targeting volatile amines in fish and other perishable foods [5,6]. This study developed a novel pH-sensitive FI designed to detect capture the pH transition associated with the fermentation of skate sashimi. Applied in the field, this FI can facilitate quality assurance, traceability, and logistics management. It may also help prevent consumer complaints or legal disputes, while providing usability and visibility to both suppliers and consumers [7].

The developed FI is sensitive to amines such as ammonia, trimethylamine (TMA), and dimethylamine (DMA). It offers an efficient approach for assessing the freshness and fermentation degree of sashimi by measuring amine concentrations. The accumulation of these volatile amine gases leads to an increase in pH. Sashimi is prone to rapid freshness loss due to various physicochemical and microbiological processes, which generate odorous compounds during protein decomposition. Traditional methods for evaluating fish freshness include chemical analysis, microbiological assays, pH measurements and total volatile basic nitrogen (T-VBN) assessment [8]. Although reliable, these methods are time-consuming, costly and impractical for routine use. In contrast, the FI emerges as a more efficient and reliable approach for evaluating skate sashimi’s freshness.

Skates have a distinct flavor and have been widely used in various dishes in the Jeolla-do Province of Korea since ancient times. They are prepared in diverse ways, including raw, steamed, boiled, and stir-fried forms. Among these, skate sashimi is consumed in its unfermented state in dishes such as sashimi [9]. Skates contain high levels of urea and urea precursors that regulate osmosis during their lifetime. During fermentation, these compounds are converted into ammonia and TMA, contributing to the characteristic flavor. Skates also have a naturally high pH, and their skin contains an antimicrobial peptide, kenojeinin I. This peptide inhibits the growth of microorganisms such as *Bacillus subtilis* and *Escherichia coli*. As a result, the surface of skate skin is not conducive to harmful bacterial survival and resists spoilage. This explains why skate is considered a fermented food. However, previous studies have reported that the edible parts of skate lack antimicrobial properties. Once the skin is removed, skate sashimi may pose a risk of harmful bacterial contamination. Thus, further research is needed to evaluate the freshness and fermentation degree of skate sashimi [10,11].

Numerous studies have investigated fermented skate. However, studies investigating indicators to evaluate the freshness and fermentation degree of skate sashimi are limited. In particular, there is a lack of research quantitatively analyzing specific volatile amines such as monomethylamine (MMA), DMA, and TMA using GC-FID. Previous studies have largely relied on T-VBN as a general freshness parameter. However, T-VBN cannot distinguish which individual amines play the most critical role in spoilage. This limitation makes it difficult to identify a clear target for freshness indicators. To address this gap, the present study aimed to develop a pH-sensitive FI capable of visually tracking skate sashimi quality decline through color change. The developed FI was evaluated under refrigerated storage by analyzing its correlations with pH, total bacterial count (TBC), T-VBN, and TMA levels quantified by GC-FID. The results of this study are expected to provide valuable insights into effective freshness indicator development. They may also contribute to the advancement of intelligent packaging systems for seafood products.

## 2. Materials and Methods

### 2.1. Materials and Chemicals

Acetone and 2-butanone were supplied by Junsei Chemical Co., Ltd. (Tokyo, Japan). Cellulose acetate was obtained from Daejung Chemicals & Metals Co., Ltd. (Seoul, Republic of Korea). Dibutyl phthalate was purchased from Sigma-Aldrich Co. (St. Louis, MO, USA). Bromothymol blue sodium salt (BTB) and bromocresol purple (BCP) were obtained from Tokyo Chemical Industry Co., Ltd. (Tokyo, Japan) and Samchun Pure Chemical Co., Ltd. (Pyeongtaek, Republic of Korea), respectively.

Industrial filter paper (HC-50) was supplied by Hyundai Micro., Ltd. (Seoul, Republic of Korea), and BRN-A120E20-L was obtained from Nitto Denko Corp. (Osaka, Japan). PDMS/DVB SPME fiber (65 µm film thickness) was supplied by Supelco (Bellefonte, PA, USA). Sodium hydroxide (NaOH) was purchased from Daejung Chemicals & Metals Co., Ltd. (Siheung, Republic of Korea). DMA hydrochloride and TMA hydrochloride were purchased from Sigma-Aldrich Co. Propylamine hydrochloride, used as an internal standard (IS), was from Tokyo Chemical Industry Co., Ltd.

### 2.2. Fabrication of the FI

The FI for assessing the fermentation level of skate was prepared as follows. A mixture of acetone and 2-butanone in a 1:1 ratio (*v*/*v*) was prepared by combining 40 mL in a flask. For the formation of the polymer matrix, 1.2 g of cellulose acetate was added and completely dissolved. BTB and BCP were then added in a 1:1 ratio, each at a quantity of 20 mg. To enhance the air sensitivity of the pH dye mixture, 0.6 mL of dibutyl phthalate was also added as a plasticizer [12].

The prepared solution was used to coat industrial filter paper (HC-50) for 30 min, followed by drying in a dark room for 2 h. The dried paper was cut into 1.8 × 1.8 cm pieces and attached to a porous breathable sheet (BRN-A120E20-L) using 2 × 2 cm customized double-sided tape (3M™, Elyria, OH, USA). A PET film was then affixed as a protective cover layer to shield against physical and chemical influences from the external environment [13].

### 2.3. Packaging of the Skate Samples

**Figure 1:**
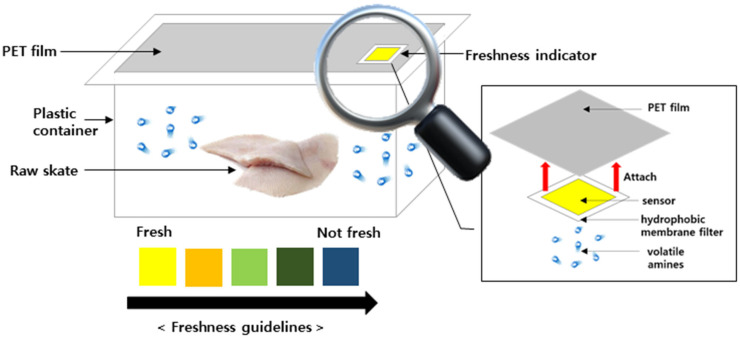
Prototype freshness indicator for skate sashimi spoilage during storage.

Skate (*Zearaja chilensis*, USA) was used as the sample in this study. The American skate was imported in a frozen state and purchased as sashimi from a local market in Mokpo, Republic of Korea in January 2024. Frozen skate samples were thawed at 4 °C until the core temperature reached 4 °C, in accordance with refrigerated thawing conditions prior to analysis. Figure 1. shows that 500 g of skate sashimi prior to fermentation was placed in a polypropylene (PP) plastic container (16 × 11 × 6 cm). The FI was applied for 7 days at 4 °C.

### 2.4. Determination of Chromaticity Value on the FI in Skate Packaging

The chromaticity of the FI was measured using a colorimeter (CR-10 Plus, Konica Minolta, Osaka, Japan) to assess its correlation with chemical and microbiological changes over 7-day storage period. Prior to each measurement, the instrument was calibrated using a standard white calibration plate to ensure measurement accuracy. All readings were performed in triplicate under consistent ambient lighting conditions, and the results are presented as mean values with corresponding standard deviations. To evaluate its practical applicability for monitoring fermentation level and quality, the FI was attached to the inner side of the packaging film of the plastic container holding the sample.

The Hunter color parameters L* (lightness), a* (red/green), and b* (blue/yellow) were measured in triplicate for each sample. L* ranges from 0 to 100, while a* (green to red) and b* (blue to yellow) range from −120 to 120 [14]. The total chromaticity difference (Δ*E*) was calculated as follows:

(1) $$\Delta E=\;\sqrt{{\left(L-L^{'}\right)}^{2}+{\left(a-a^{'}\right)}^{2}+{\left(b-b^{'}\right)}^{2}}$$

In addition, prior to application, the colorimetric response of the FI was characterized using Britton–Robinson buffer solutions ranging from pH 5.0 to 10.0 at 25 °C. The FI were contacted with each buffer, and the resulting colors were recorded under controlled lighting conditions. The Hunter color parameters L*, a*, and b* were measured for FI at each pH point from 5.0 to 10.0, and the total color difference (Δ*E*) relative to pH 5.0 was calculated in triplicate. These measurements defined the operational pH window and confirmed the suitability of the indicator mixture for detecting spoilage-related pH changes.

### 2.5. Measurement of T-VBN in Skate During Storage

The content of T-VBN was quantified using the microdiffusion method with Conway units. A 5 g sample was homogenized with 25 mL of distilled water for 90 s using a homogenizer (HG-15D, Daehan Scientific Co., Ltd., Seoul, Republic of Korea). The homogenate was centrifuged at 3000 rpm for 15 min. The supernatant was transferred to a 25 mL volumetric flask and diluted to volume with water.

The diluted solution (1 mL) was placed in the outer part of the Conway dish. The inner part received 1 mL of 1% H_3_BO_3_ and 10 µL of Conway reagent, consisting of 0.66% methyl red in ethanol and 0.66% bromocresol green in ethanol. Saturated potassium carbonate solution (1 mL, K_2_CO_3_) was then added to the outer part.

The dish was incubated at 25 °C for 90 min (BI-150, Hanyang Scientific Equipment Co., Ltd., Seoul, Republic of Korea). After incubation, the sample was titrated with 0.02 N hydrochloric acid (HCl) until the Conway reagent color changed from blue to red [15]. The T-VBN values were calculated using the following formula:

(2) $$T-VBN\left(mg\%/100g\right)=\frac{14.007\times \left(x-y\right)\times f\times 100\times d}{S}$$

Here, *x* and *y* represent the titration volumes (mL) for the sample and the blank, respectively. *f* is the equivalent factor of HCl. *S* is the sample weight (g), and *d* is the dilution factor.

### 2.6. Measurement of pH Value in Skate During Storage

The sample was prepared in the same way as described in Section 2.5. The pH of USA skate slices was measured using a pH meter (Orion Star TM A211 Conductivity Benchtop Meter; Thermo Fisher Scientific, Waltham, MA, USA) [16].

### 2.7. Counting of Total Bacteria in Skate Using the Petrifilm Plates During Storage

Microbial analysis followed the AOAC-approved method listed in the FDA Bacterial Analysis Manual [14]. Skate samples (10 g) were serially diluted with 90 mL of sterile saline solution. The samples were blended in a stomacher bag using a BagMixer^®^ 400 W (Interscience, Puycapel, Cantal, France) at 20 °C for 4 min.

A 1 mL portion of the mixture was pipetted onto 3M™ aerobic count Petrifilms and incubated at 35 ± 1 °C for 48 ± 2 h. TBC were expressed as colony-forming units per gram (log CFU/g) [17].

### 2.8. Headspace-Solid-Phase Microextraction (HS-SPME) Analysis

Solid-phase microextraction (SPME) was introduced by Pawliszyn as an extraction technique that combines sampling and sample preparation in one step [18]. In this method a fused silica fiber coated with a polymeric stationary phase is placed in a headspace vial to quantify three types of amines [19]. Amine stock solutions (1000 µg/mL) were prepared in water and diluted to 50, 100, 250, 500, and 750 µg/mL for 5-point calibration curves. A micromagnetic stirrer bar and 10 µg/mL IS were placed in a 20 mL vial [20]. Each standard solution (MMA, DMA, TMA, and IS; 1 mL) and saturated NaCl solution 1 mL was added to the vial, followed by 5 mL of 40% (*v*/*v*) NaOH solution [21]. The vials were stirred while the SPME fiber was exposed to the headspace at 25 °C for 20 min, allowing volatile amines to reach equilibrium and be simultaneously extracted. After extraction the fiber was inserted into the GC injector port for 5 min desorption, followed by GC-FID analysis [22].

A 5 g sample of USA skate slices was finely cut, and 5 mL of 2% (*v*/*v*) trichloroacetic acid (TCA) solution was added. The mixture was allowed to stand for 10 min. The sample was then combined with 25 mL of water and homogenized at 1500 rpm for 1 min. The homogenate was centrifuged at 3000 rpm for 15 min. From the supernatant, 1 mL was transferred to a 20 mL vial with 2 mL of water, replacing the MMA, DMA, and TMA standards. This mixture was analyzed using the procedure described in the previous paragraph.

### 2.9. Analysis of MA, DMA, and TMA Using GC-FID

Volatile amines in the samples were identified by differences in retention time according to the molecular weights of MMA, DMA, TMA, and IS. Peak height was compared with calibration standard concentrations to confirm identity. Limits of detection (LOD) and limits of quantification (LOQ) were determined as previously described. LOQ was defined as the lowest concentrations within the linear range. *LOD* and *LOQ* values were calculated using the following formula:

(3) $$LOD=3.3\;\times \frac{\delta }{S},\;\;LOQ=10\;\times \;\frac{\delta }{S}\;$$

Here, *σ* represents the standard deviation of blank measurements, and *S* is the slope of the calibration curve.

The analysis was performed using an Agilent Technologies 7890A gas chromatograph (Santa Clara, CA, USA) equipped with a flame ionization detector. Amines were separated on a CP-Volamine capillary column (30 m × 0.32 mm × 0.25 µm; J&W Scientific, Folsom, CA, USA). The GC conditions were as follows. Injector temperature was 250 °C. The injection mode was splitless, and the liner type was an ultra-inert inlet liner straight, SPME taper, 0.75 mm. Nitrogen was used as the make-up gas at a flow rate of 40 mL/min. The air flow rate was 400 mL/min and the hydrogen flow rate was 40 mL/min. The detector temperature was maintained at 300 °C. The oven temperature was initially set at 40 °C for 5 min, then increased at 10 °C/min to 150 °C, and held for 10 min [23].

### 2.10. Statistical Analysis

All experiments were conducted in triplicate. Results are expressed as mean ± standard deviation. One-way ANOVA was used to determine significant differences at *p* < 0.05, followed by Duncan’s test. Pearson correlation analysis was performed for pH, VBN, amines, and TBC. Statistical analyses were conducted using SPSS software version 27.0 (SPSS Inc., Chicago, IL, USA).

## 3. Results and Discussion

### 3.1. The Response of the FI to Skate Slices During Storage

Prior to application in skate sashimi packaging, the FI was evaluated using Britton–Robinson buffers (pH 5.0–10.0). The FI exhibited clear and progressive color changes across the tested pH values. The Hunter parameters shifted systematically with pH, and the Δ*E* values increased consistently. The chromaticity values are summarized in Table 1. These results defined the operational pH window of the FI. These preliminary results confirmed that the FI was responsive to alkaline pH changes relevant to volatile amine release during spoilage.

The chromatic responses of the FI across pH 5–10 are summarized in Table 1. Significant differences (*p* < 0.05) were observed in Δ*E*, L*, a*, and b* values among all pH groups. Δ*E* values increased progressively with pH, confirming gradual and distinguishable color differentiation. L* decreased with higher pH, reflecting a loss of lightness, while a* and b* values shifted toward the green and blue axes, respectively. These results demonstrate that the FI exhibited predictable and systematic chromatic transitions under controlled pH conditions, thereby validating its applicability for detecting alkaline shifts associated with volatile amine release.

The FI was attached to the bottom of the film inside the skate sashimi packaging. It detected the gradual release of VBN (e.g., MMA, DMA, TMA, and NH_3_), which led to an increase in pH [16]. In response to VBN accumulation the FI exhibited progressive color changes as shown in Figure 1. Therefore, analyzing the headspace gas in the packaging was an essential part of this study. Skate sashimi was stored at 4 °C to simulate typical household refrigeration conditions.

Over the 7-day storage period, distinct color changes were observed in the FI. The color shifted from the initial yellow to the final blue in response to variations in quality measurement indicators. These visual observations were supported by significant changes in chromaticity values, see Table 2.

Δ*E* increased from day 2 (7.72 ± 0.01) to day 3 (23.52 ± 0.13), indicating a transition in chromaticity. The rate of Δ*E* change between days 3 and 5 was approximately 11.53 units/day, significantly higher than at other intervals. This shift corresponded to the visual change in the indicator from yellow to bluish-green. By day 7, the indicator had turned distinctly blue. The color shift was consistent with changes in the Hunter parameters. L* decreased from 75.20 to 49.63. The a* value dropped from 3.20 to −0.50. The b* value shifted from 41.70 to −7.37, indicating a clear trend toward the blue-green axis.

The chromatic changes suggest that the breakdown of trimethylamine oxide (TMAO) into TMA began around day 2 and intensified through day 5. The slower rate of change thereafter may reflect depletion of precursors. Stability under ambient light and prolonged storage confirms the FI’s durability. The association between color transition and pH variation in the package headspace indicates that the FI’s response was driven by a pH-sensitive chemical reaction under alkaline conditions.

### 3.2. The pH Measurement in Skate Sashimi During Storage

The pH of fish typically decreases postmortem due to the anaerobic breakdown of glycogen. This process generates lactic acid and lowers the pH to around 5.4. It then increases again during autolysis and the formation of alkaline substances, eventually reaching the point of spoilage. In white-fleshed fish the pH generally ranges from 6.7 to 6.8. In red-fleshed fish it is around 6.2 to 6.4 [24]. In contrast, the pH of skates is notably alkaline compared with other fish. This is due to their classification as cartilaginous fish such as sharks and rays, which contain substantial amounts of osmoregulatory compounds like TMAO. During processing, distribution, and storage these compounds are converted into alkaline substances, including NH_3_, TMA, and DMA [25].

**Figure 2:**
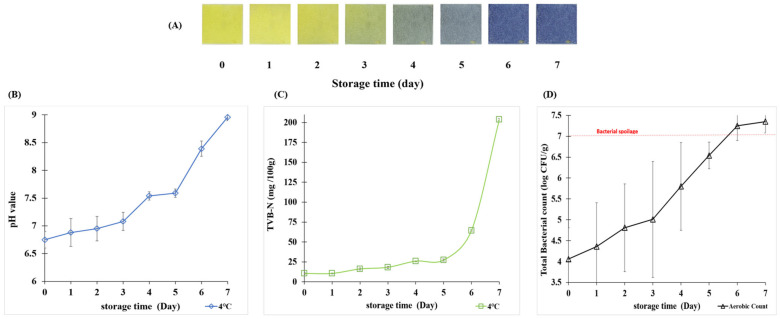
Changes in color (**A**); pH (**B**); T-VBN (**C**); and TBC (**D**) content of skate during storage at 4 °C.

pH is defined in aqueous solutions as the concentration of protons (H^+^), and cannot be strictly applied to gases. However, the high humidity inside food packaging promotes the formation of a thin water layer on the FI surface. In this layer, volatile amines become protonated and trigger color changes in pH-sensitive indicators. Previous studies have confirmed that volatile amines can be detected in the gas phase using smart-material-based indicators that exhibit clear color transitions upon amine exposure [26]. The pH of the skate was 6.75 in its initial fresh state. Over the 7-day storage period it gradually increased to 8.96. This supports the conclusion that microbial activity in skate leads to the breakdown of TMAO into TMA and NH_3_, resulting in a pH increase [10,27]. The pH change pattern is shown in Figure 2B [28]. This upward trend matched the color transition observed in the pH-sensitive FI developed. During days 1–2 the FI maintained yellow, reflecting a relatively low pH (≤6.7). The pH exceeded 7.0 between days 3 and 4 and the indicator shifted to green or greenish-blue. From day 5 onward the pH rose sharply above 8.0 and the FI color changed to blue, visually indicating the onset of freshness loss. Furthermore, the FI labels themselves showed no significant change under ambient lighting and remained stable for more than 3 months without detectable variation.

The observed color transitions of the FI were associated with pH variations in the headspace. This indicates that the color change was driven by a pH-sensitive chemical response. The FI was prepared by mixing BTB and BCP at a 1:1 ratio. BCP has a pKa of 6.3, with a transition between pH 5.2–6.8, while BTB has a pKa of 7.1, with a transition between pH 6.0–7.6. The overlap between these intervals (6.0–6.8) suggested the potential for an extended response range, which was experimentally verified. This functional pH range closely matched the shift observed in skate sashimi during storage (6.7–9.0), confirming the suitability of the FI for real-time monitoring of seafood freshness.

### 3.3. T-VBN Measurement in Skate Sashimi During Storage

T-VBN reflects the accumulation of volatile nitrogen-containing compounds which influence pH and trigger color changes in the FI. It serves as a chemical basis for evaluating freshness. T-VBN is widely used as a standard parameter to assess fish and seafood quality, most commonly through Conway’s microdiffusion method. In fresh fish, the T-VBN level typically ranges from 5 to 20 mg N/100 g of muscle [29].

However, the naturally high T-VBN content in skate made the standard Conway method difficult to apply directly. H_3_BO_3_ was added to the Conway dish to enable proper titration. Using this adjusted method, the T-VBN value of skate sashimi was 10.5 mg N/100 g on day 0, indicating freshness comparable to raw fish. T-VBN remained stable until day 3, then increased from day 3 to day 5. During this period the FI shifted from yellow to green. From day 5 to day 7 T-VBN rose sharply to 64.4 and 203.7 mg N/100 g, as shown in Figure 2B.

This increase likely reflects intensified microbial activity and the breakdown of TMAO into TMA and NH_3_. The timing of this change matched the FI’s transition from green to blue, indicating a strong link between chemical and visual freshness indicators. The results align with previous reports. For example, Lee (1999) reported that the T-VBN content in fermented skate ranged from 36 to 260 mg N/100 g depending on sampling season, degree of ripeness, and processing methods [30]. Despite differences in fermentation status and storage conditions, the values obtained here fall within this range. This agreement suggests that the analytical approach including the modified Conway method provides reliable measurements for skate sashimi. It also supports the validity of T-VBN as a comparative parameter when interpreting freshness-related chemical changes in this species.

### 3.4. GC-FID Quantification Method

While T-VBN is widely used as a standard indicator for assessing fish freshness it represents only the overall concentration of nitrogenous compounds. It does not identify the specific volatile amines responsible for quality deterioration or FI color changes. This limitation makes it difficult to interpret chemical changes during storage with precision. To address this GC-FID analysis was used to quantify individual volatile amines such as MMA, DMA, and TMA in skate sashimi.

Calibration curves were prepared for each amine at five concentrations (5, 10, 25, 50, and 75 µg/kg). All curves showed excellent linearity with coefficients of determination (*R*^2^) greater than 0.99. The regression equations were *y* = 0.0013*x* + 0.0024 (*R*^2^ = 0.9987) for MMA, *y* = 0.1333*x* + 0.0845 (*R*^2^ = 0.9915) for DMA, and *y* = 0.0156*`* + 0.0249 (*R*^2^ = 0.9985) for TMA. The LOD were 4.10 mg/kg for MMA, 0.51 mg/kg for DMA, and 5.30 mg/kg for TMA. The LOQ were 12.41, 1.54, and 16.07 mg/kg. Recoveries were obtained from the calibration standards. The values were 91.5–103.8% for MMA, 92.0–111.9% for DMA, and 76.0–108.5% for TMA. These values are summarized in Table 3 [23].

Although many studies have measured T-VBN in skate most used Conway’s microdiffusion method and did not isolate individual amines. No previous research has quantified MMA, DMA, and TMA in skate sashimi using GC-FID. This work therefore offers new insight by applying a compound-specific analytical approach.

### 3.5. Analysis of MMA, DMA, and TMA Using GC-FID

**Figure 3:**
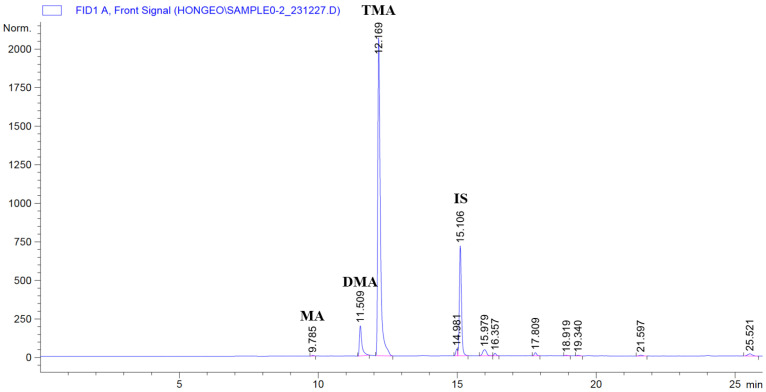
GC-FID chromatogram of volatile amines of a sashimi sample.

GC-FID analysis was performed to identify and quantify volatile amines related to freshness loss in skate sashimi. The target compounds were MMA, DMA, and TMA. Figure 3. presents a representative chromatogram of a skate sashimi sample containing MMA, DMA, TMA, and IS. Identification was determined from the retention times of pure standards: MMA (9.75–9.80 min), DMA (11.05–11.55 min), TMA (12.20–12.42 min), and IS (15.10–15.52 min).

Table 4 shows that MMA was not detected during the 7-day storage period and remained below the limit of detection. DMA was present at low levels (0.84–1.23 mg/kg) and did not change significantly over time. TMA rose sharply from 6.84 mg/kg on day 3 to 263.37 mg/kg on day 5. It stayed above 200 mg/kg through day 7. This increase is consistent with intensified microbial activity and the enzymatic reduction of TMAO to TMA, a major cause of the characteristic odor and freshness loss in marine fish. The timing of this change matched the FI color shift from yellow to blue, confirming the link between chemical indicators and visual freshness assessment.

Previous studies have reported a gradual increase in TMA-N over time when using the Conway microdiffusion method [19]. Although GC-FID with HS-SPME was applied in the present study, the overall trend in TMA accumulation was in agreement with these earlier findings. TMA concentrations in commercially available fermented skate have been reported at 110.9–150.1 µg/mL, which is comparable to the levels observed here during the later stages of storage [10]. This agreement suggests that GC-FID not only serves as a reliable quantitative tool but also provides enhanced specificity by distinguishing among individual volatile amines.

### 3.6. Microbiological Assessment of Skate Sashimi During Storage

Skate flesh contains substantial amounts of TMAO, an osmoregulatory compound. During storage TMAO is broken down by microbial activity, producing odorous compounds such as TMA and NH_3_ that contribute to the characteristic smell of skate [31]. This study examined TBC in skate sashimi over 7 days to better understand the relationship between microbial growth and the release of these volatile amines.

Figure 2D shows that TBC in skate slices ranged from 4.06 to 7.35 log CFU/g during storage. From day 0 to day 3 TBC remained stable, indicating a lag phase in microbial growth. A rapid increase occurred between days 3 and 6, marking an exponential growth phase. By day 5 TBC reached about 10^6^–10^7^ CFU/g, a level often regarded as the spoilage threshold for fishery products. From day 6 onward the growth rate slowed, suggesting that microbial populations had entered the stationary or early death phase.

These trends align with previous findings. S.H. Cho et al. (2006) reported that microbial load on skate skin increased from 4 log CFU/g before fermentation to 7 log CFU/g after fermentation [32]. This supports the pattern observed here where a sharp TBC increase from day 3 to day 6 corresponded with FI color change, reinforcing the link between microbial activity and visual freshness indicators.

### 3.7. The Correlation Statistical Analysis Between pH, TBC, T-VBN, TMA, and ΔE

To identify which freshness-related parameters most strongly affect FI color change, Pearson correlation analysis was conducted using Δ*E* values and quality indicators during storage, see Table 5.

Δ*E* showed positive correlation with TBC (*r* = 0.978, *p* = 0.000), pH (*r* = 0.901, *p* = 0.002) and TMA (*r* = 0.888, *p =* 0.003). The correlation with T-VBN was lower (*r* = 0.654, *p* = 0.078) and not statistically significant.

The strong correlation with TBC suggests that microbial growth influenced FI color change through metabolic byproducts. The high correlation with pH indicates that increased alkalinity in the headspace was closely linked to color shifts in the indicator. The correlation with TMA reflects its role as a major volatile amine produced from the microbial reduction of TMAO, contributing to freshness loss and blue coloration of the FI. The weak correlation with T-VBN (*r* = 0.654) can be explained by differences in protonation behavior among volatile basic compounds. Although TMA (pKa ~ 9.8) and NH_3_ (pKa ~ 9.25) have similar pKa values, their protonation efficiencies differ significantly. At 298 K, the base dissociation constants (Kb) of DMA (aq) and TMA (aq) are reported to be 31-fold and 4-fold higher than that of NH_3_ (aq), respectively [33]. As a result, TMA is more readily protonated in the thin aqueous layer on the FI surface and strongly drives the color change, whereas NH_3_ contributes less. This mechanistic difference explains why ΔE correlates with TMA, while the inclusion of NH_3_ in T-VBN measurements weakens the overall correlation. In future studies, a quantitative evaluation of NH_3_ would allow the relative contributions of individual compounds to be clarified more precisely. These findings support the inclusion of TBC, pH, and TMA as key parameters for FI calibration to improve freshness assessment for skate sashimi under cold storage.

## 4. Conclusions

This study developed a pH-sensitive freshness indicator (FI) for skate sashimi (*Zearaja chilensis*) targeting volatile amines, primarily TMA. The FI exhibited a clear yellow-to-blue color transition over 7 days at 4 °C, aligning with changes in pH, TMA, T-VBN, and TBC. Colorimetric parameters showed strong correlations with pH (*r* = 0.901), TMA (*r* = 0.888), and TBC (*r* = 0.978), confirming the FI’s effectiveness as a visual and quantitative freshness sensor.

The indicator responded reliably despite the high baseline VBN content typical of skate, indicating robustness in complex matrices. It offers a practical tool for real-time monitoring of seafood freshness during storage. For broader application to other sashimi species, tuning the pH sensitivity by pre-treatment with HCl or NaOH may be required to match species-specific spoilage profiles [34].

## Figures and Tables

**Table 1:** The chromaticity values of the FI mixture measured in Britton-Robinson buffers.

Color Value	pH Value and Color Change						
pH	Control	5	6	7	8	9	10
	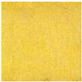	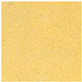	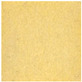	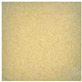	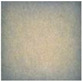	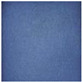	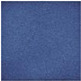
**Δ*E***	$0.00^{\;a}$	${10.57\pm 0.13}^{\;b}$	${8.70\pm 0.15}^{\;c}$	${15.61\pm 0.12}^{\;d}$	${57.50\pm 0.13}^{\;e}$	${90.34\pm 0.42}^{\;f}$	${89.20\pm 1.01}^{\;g}$
***L***	${75.23\pm 0.06}^{\;a}$	${68.50\pm 0.35}^{\;b}$	${67.37\pm 0.06}^{\;c}$	${58.37\pm 0.12}^{\;d}$	${37.10\pm 0.10}^{\;e}$	${31.53\pm 0.06}^{\;f}$	${31.27\pm 0.38}^{\;f}$
***a***	$1{3.40\pm 0.00}^{\;a}$	${19.23\pm 0.06}^{\;b}$	${17.07\pm 0.15}^{\;c}$	${13.30\pm 0.10}^{\;c}$	${9.17\pm 0.38}^{\;d}$	${2.03\pm 0.15}^{\;e}$	${0.77\pm 0.55}^{\;f}$
***b***	$59.90{\pm 0.00}^{\;a}$	$67.83{\pm 0.12}^{\;b}$	${66.03\pm 0.21}^{\;c}$	${52.93\pm 0.15}^{\;d}$	${14.20\pm 0.17}^{\;e}$	${-19.90\pm 0.50}^{\;f}$	${-18.27\pm 1.02}^{\;g}$

(1) All values are mean ± standard deviation of triplicate determination. (2) Means with different superscript in the same row are significantly different (*p* < 0.05) based on the Duncan test. (3) Control corresponds to the FI without buffer treatment and is shown as 0 for comparison.

**Table 2:** The chromaticity value changes in the indicator response during storage (4 °C, 7 days).

Color Value	Storage Time and Color Change (Day)							
	0	1	2	3	4	5	6	7
	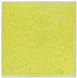	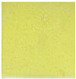	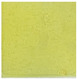	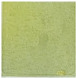	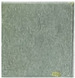	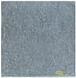	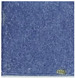	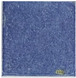
**Δ*E***	$0.00^{\;a}$	${0.76\pm 0.07}^{\;b}$	${7.72\pm 0.01}^{\;c}$	${23.52\pm 0.13}^{\;d}$	${38.28\pm 0.10}^{\;e}$	${45.59\pm 0.05}^{\;f}$	${52.50\pm 0.04}^{\;g}$	${55.45\pm 0.23}^{\;h}$
***L***	${75.20\pm 0.00}^{\;a}$	${74.60\pm 0.10}^{\;b}$	${71.40\pm 0.20}^{\;c}$	${63.10\pm 0.20}^{\;d}$	${57.00\pm 0.10}^{\;e}$	${54.33\pm 0.06}^{\;f}$	${51.77\pm 0.06}^{\;g}$	${49.63\pm 0.29}^{\;h}$
***a***	${3.20\pm 0.00}^{\;a}$	${2.90\pm 0.10}^{\;b}$	${0.87\pm 0.06}^{\;c}$	${-2.17\pm 0.06}^{\;e}$	${-2.27\pm 0.06}^{\;f}$	${-1.50\pm 0.00}^{\;d}$	${-0.57\pm 0.06}^{\;c}$	${-0.50\pm 0.00}^{\;c}$
***b***	$41.70{\pm 0.00}^{\;a}$	${41.77\pm 0.42}^{\;a}$	${35.40\pm 0.10}^{\;b}$	${22.70\pm 0.06}^{\;c}$	${8.47\pm 0.06}^{\;d}$	${1.43\pm 0.06}^{\;e}$	${-5.1\pm 0.06}^{\;f}$	${-7.37\pm 0.12}^{\;g}$

(1) All values are mean ± standard deviation of triplicate determination. (2) Means with different superscript in the same row are significantly different (*p* < 0.05) based on the Duncan test.

**Table 3:** The LOD, LOQ, Linearity equation, *R*^2^, and Recovery of the three volatile basic amines.

Volatile Basic Amines	LOD ^a^ (mg/kg)	LOQ ^b^ (mg/kg)	Linearity Equation ^c^	*R* ^2^	Recovery (%)
MMA	4.0959	12.4119	*y* = 0.0013*x* + 0.0024	0.9987	91.5–103.8
DMA	0.5078	1.5386	*y* = 0.1333*x* + 0.0845	0.9915	92.0–111.9
TMA	5.3021	16.0669	*y* = 0.0156*x* + 0.0249	0.9985	76.0–108.5

^a^ set up in a signal-to-noise ratio (S/N) = 3.3; ^b^ set up in a signal-to-noise ratio (S/N) = 10; ^c^ numbers express mean values (*n* = 3).

**Table 4:** Quantitative determination of simultaneous analysis of MMA, DMA, and TMA during the storage (4 °C, 7 days).

Volatile Amine	Storage Time (Day)							
	0	1	2	3	4	5	6	7
MMA (mg / kg)	N.D.	N.D.	N.D.	N.D.	N.D.	N.D.	N.D.	N.D.
DMA (mg / kg)	${0.99\;\pm \;0.45\;}^{\;c}$*	${0.87\;\pm \;0.38\;}^{\;b}$*	${1.11\;\pm \;0.37}^{\;\;f}$*	${1.05\;\pm \;0.53\;}^{\;d}$*	${0.84\;\pm \;0.37\;}^{\;a}$*	${1.20\;\pm \;0.38}^{\;h}$*	${1.23\;\pm \;0.28\;}^{\;g}$*	${1.05\;\pm \;0.39\;}^{\;e}$*
TMA (mg / kg)	${5.46\;\pm \;0.28\;}^{\;a}$*	${5.67\pm \;0.88\;}^{\;a}$*	${6.84\;\pm \;0.73\;}^{\;b}$*	${10.47\;\pm \;0.38\;}^{\;c}$*	${84.75\;\pm \;0.58\;}^{\;d}$	${263.37\pm \;0.28\;}^{\;g}$	${204.66\;\pm \;0.52\;}^{\;f}$	${202.26\;\pm \;0.38\;}^{\;e}$

(1) All values are mean ± standard deviation of triplicate determination. (2) Means with different superscript in the same row are significantly different (*p* < 0.05) based on the Duncan test. (3) N.D.: Not Detected, the lower limit of detection. (4) *: The asterisk (*) indicates values between the *LOD* and the *LOQ*.

**Table 5:** Correlation between the color indicator towards pH, TBC, T-VBN, TMA values (4 °C, 7 days).

Parameter	Δ*E*	*L*	*a*	*b*	pH	TBC	T-VBN	TMA
Δ*E*	1 ***	−0.999 ***	−0.720 *	−1.000 ***	0.901 **	0.978 ***	0.654	0.888 **
*L*	−0.999 ***	1 ***	0.763 *	0.997 ***	−0.891 **	−0.970 ***	−0.651	−0.870 **
*a*	−0.734 *	0.763 *	1 ***	0.720 *	−0.441	−0.616	−0.202	−0.481
*b*	−1.000 ***	0.997 ***	0.720 *	1 ***	−0.905 **	−0.981 ***	−0.658	−0.895 **

* Statistically significant at *p* < 0.05, ** statistically significant at *p* < 0.01, *** statistically significant at *p* < 0.001.

## Data Availability

The data presented in this study are available on request from the corresponding author.
